# High Levels of MeCP2 Depress MHC Class I Expression in Neuronal Cells

**DOI:** 10.1371/journal.pone.0001354

**Published:** 2007-12-26

**Authors:** Julie Miralvès, Eddy Magdeleine, Lara Kaddoum, Hélène Brun, Sophie Peries, Etienne Joly

**Affiliations:** 1 Institut de Pharmacologie et Biologie Structurale, Centre National de la Recherche Scientifique (CNRS), Toulouse, France; 2 Centre de Physiopathologie Toulouse-Purpan, INSERM, Toulouse, France; Northwestern University, United States of America

## Abstract

**Background:**

The expression of MHC class I genes is repressed in mature neurons. The molecular basis of this regulation is poorly understood, but the genes are particularly rich in CpG islands. MeCP2 is a transcriptional repressor that binds to methylated CpG dinucleotides; mutations in this protein also cause the neurodevelopmental disease called Rett syndrome. Because MHC class I molecules play a role in neuronal connectivity, we hypothesised that MeCP2 might repress MHC class I expression in the CNS and that this might play a role in the pathology of Rett syndrome.

**Methodology:**

We show here that transiently transfected cells expressing high levels of MeCP2 specifically downregulate cell-surface expression of MHC class I molecules in the neuronal cell line N2A and they prevent the induction of MHC class I expression in response to interferon in these cells, supporting our first hypothesis. Surprisingly, however, overexpression of the mutated forms of MeCP2 that cause Rett syndrome had a similar effect on MHC class I expression as the wild-type protein. Immunohistological analyses of brain slices from *MECP2* knockout mice (the MeCP2^tm1.1Bird^ strain) demonstrated a small but reproducible increase in MHC class I when compared to their wild type littermates, but we found no difference in MHC class I expression in primary cultures of mixed glial cells (mainly neurons and astrocytes) from the knockout and wild-type mice.

**Conclusion:**

These data suggest that high levels of MeCP2, such as those found in mature neurons, may contribute to the repression of MHC expression, but we find no evidence that MeCP2 regulation of MHC class I is important for the pathogenesis of Rett syndrome.

## Introduction

Mutations in the X-linked gene *MECP2* cause Rett syndrome (RTT) [1]–[3], a progressive neurodevelopmental disorder that affects around 1 in 10,000 female births [4]. Girls with RTT develop normally until 6–18 months old, when they begin to regress, losing the speech and hand skills they had acquired. Most patients develop severe mental retardation, seizures, repetitive hand movements, irregular breathing and motor-control problems [5]–[7]. *MECP2* encodes the methyl-CpG-binding protein 2 (MeCP2) [8], [9], which is thought to regulate gene expression, but we do not yet understand how mutations in this protein produce the pathological features of RTT.

*MECP2* knockout mice [10]–[12] and mice expressing a truncated form of MeCP2 [13] display a RTT-like phenotype, demonstrating that MeCP2 deficiency is sufficient to induce the syndrome. Moreover, mice in which *MECP2* was conditionally deleted in neurons had a similar phenotype to RTT patients [10], [14], and MeCP2-deficient mice were cured by expression of a transgenic *MECP2* gene specifically in post-mitotic neurons [15]. These findings indicate that the pathology of RTT is due to the lack of MeCP2 in the mature central nervous system (CNS). Two recent studies have shown that the neurological defects in mutant mice are reversed by restoration of MeCP2 expression in neurons, even at late postnatal stages [16], [17], suggesting that gene therapy may be feasible.

The *MECP2* mRNA is alternatively spliced to generate two protein isoforms (MeCP2*A* and MeCP2*B*) that differ at their N-termini. Both forms are expressed ubiquitously, but MeCP2*B* is more abundant than MeCP2*A* in the CNS [18], [19]. MeCP2 represses transcription [20] by binding through its methyl-CpG-binding domain (MBD) [21] to methylated CpG nucleotides [22], [23] and recruiting co-repressors that bind to its transcription repression domain (TRD) [24]–[27]. Other studies, however, indicate that MeCP2 is a multifunctional protein that affects gene regulation at many levels: MeCP2 interacts with an RNA-binding protein called Y box-binding protein 1 to regulate splicing of target RNAs [28], [29]; two studies suggest that MeCP2 influences gene expression by participating in chromatin architecture, independently of its capacity to bind methylated DNA [30], [31]; moreover, MeCP2 associates with the DNA methyltransferase Dnmt1 bound to hemi-methylated DNA and may thus participate in maintaining the methylation state of newly synthesised DNA [32], [33].

Our knowledge of the activities of MeCP2 suggests that the pathologies associated with *MECP2* mutations are most likely due to the misregulation of neuronal genes. Several studies have identified possible target genes that are controlled by MeCP2, including the gene encoding brain-derived neurotrophic factor (BDNF) [34]–[36], genes encoding inhibitors of differentiation [37], and genes regulated by glucocorticoids [38]; their expression is altered in *MECP2* knockout mice, but whether this is responsible for the neuropathology seen in RTT remains questionable.

We hypothesised that genes encoding MHC class I molecules (MHC class I) might be amongst those that MeCP2 regulates in the CNS because these genes have a particularly high CG content [39]. Two studies have demonstrated that expression of MHC class I is dynamically regulated during the post-natal development of the CNS and that MHC class I expression is necessary for the activity-dependent synaptic rearrangements that occur during normal brain development and for normal synaptic plasticity in the mature hippocampus [40], [41]. If MHC class I expression was regulated by MeCP2, defects in these developmental functions of MHC class I might contribute to the symptoms of RTT.

MHC class I is generally not expressed in adult brain except in response to cytokines (i.e. inflammation) or injury [42]. This is believed to protect nervous tissue, which regenerates poorly, from cytotoxic attack by the immune system. Several lines of evidence, however, suggest that MHC class I is expressed in specific areas of the mature brain, as well as during brain development, and they evoke a function for MHC class I molecules in neuronal signalling [41], [43]–[46]. This suggests that, rather than being simply shut down, MHC class I genes must be very finely regulated throughout the CNS by activators and inhibitors that, on the one hand, allow their function during development and, on the other hand, ensure their silencing in the majority of neurons in which their expression could be detrimental.

We reasoned that misregulated expression of MHC class I in the brain due to mutations in MeCP2 might disturb the establishment and maintenance of neuronal connections and remodelling in the hippocampus during early child development and thus account for the neurodevelopmental disorders of RTT. We therefore investigated whether MHC class I gene expression is under the control of MeCP2 in neuronal cell lines in culture and whether MHC class I gene expression is affected in *MECP2* knockout mice.

## Results

### Overexpression of MeCP2 downregulates basal MHC class I

**Figure 1:**
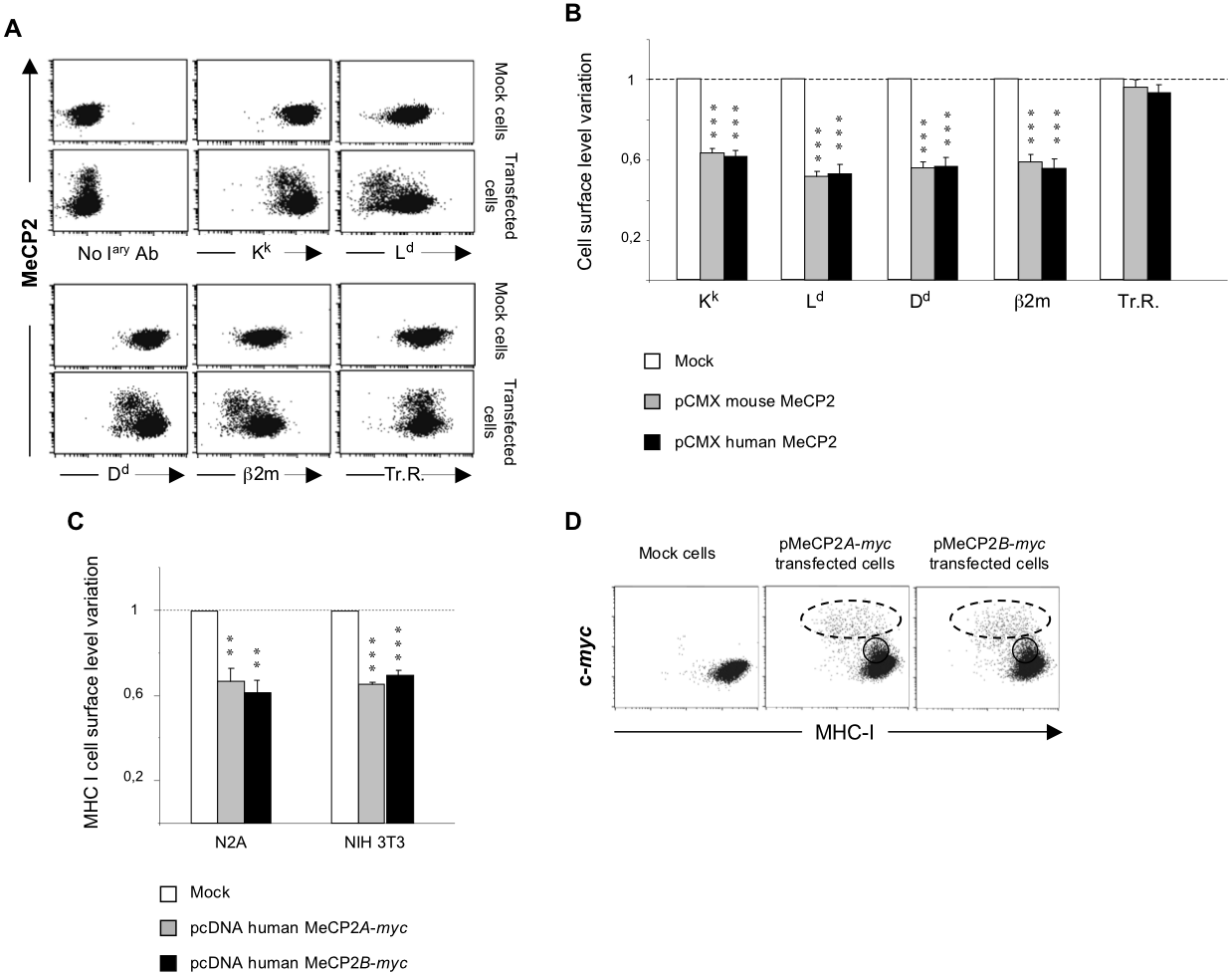
MeCP2 overexpression diminishes MHC class I expression. N2A cells transfected with pCMX vectors expressing either murine or human MeCP2 were immunostained for one of the three MHC class I molecules (K^k^, L^d^ or D^d^), β2-microglobulin or the transferrin receptor on the cell surface as well as for intracellular MeCP2. The level of staining was then analysed by fluorescence-activated cell sorting. Panel A: Dot plots of the amount of surface antigen (x-axis) against the amount of intracellular MeCP2 (y-axis) in cells transiently transfected with pCMX expressing human MeCP2 and analysed 48 hrs later. Panel B: For each kind of staining, the variation in expression level was calculated as the ratio of MFI of MeCP2 overexpressing cells over MFI of mock-transfected cells. The histograms summarise the mean (±SEM) of the variation in cell surface levels from 15 independent transfections with vectors expressing mouse MeCP2*A* (grey fill) and 12 independent transfections with vectors expressing human MeCP2*A* (black fill). Panel C: N2A and NIH3T3 cells were transfected with empty pcDNA3.1(+) (mock cells) or expressing Myc-tagged human MeCP2*A* or *B* isoforms. 48 h after transfection, cells were subjected to double staining against cell surface MHC class I molecules and intracellular Myc-tagged MeCP2, then analyzed by flow cytometry. The variation in expression level of MHC class I molecules was calculated as the ratio of MFI of MeCP2 over-expressing cells over the MFI of mock cells. The histograms represent the mean (±SEM) of the cell surface level variation from 4 independent transfections with each of the vectors. Panel D: A representative example of dot-plots obtained for double immunostaining of transiently transfected N2A cells with anti-Myc 9E10 and rat-anti-mouse-MHC I M1/42 monoclonal antibodies. Dotted and continuous circles indicate the different populations expressing high and intermediate levels of MeCP2, respectively. Statistical significance of difference between groups was analysed by using an unpaired t-test (**, *p*<0.01 ; ***, *p*<0.001).

To investigate whether MeCP2 represses MHC class I expression, we transfected the murine neuronal cell line N2A with pCMX plasmids that transiently express either human or murine MeCP2*A*. Forty-eight hours after transfection, we evaluated by flow cytometry the cell surface levels of K^k^, L^d^ and D^d^ (the three ‘classical’ MHC class I molecule heavy chains expressed by N2A), *β-*2-microglobulin (the light chain subunit of MHC class I) and, as control, the transferrin receptor, a cell surface glycoprotein of similar size and half-life to MHC molecules (Figure 1). The expression of MeCP2 in transfected cells was detected by a rabbit anti-MeCP2 polyclonal antibody on cells fixed and permeabilised after they had been stained for MHC class I cell surface expression. Dot plot representative examples of these analyses are shown on Figure 1A, where each dot represents an individual cell, and an decrease in MHC staining results in a shift to the left, and an increase in MeCP2 in an upward shift. Because these are transient transfections, MeCP2 overexpression is detected in only a certain percentage (typically 20–30%) of the cells. As can be seen for all MHC class I molecules ( K^k^, L^d^, D^d^ ) and *β*-2-microglobulin, in the cloud of cells overexpressing MeCP2, we detected a clear shift to the left compared to the lower cloud of untransfected cells and to mock-transfected cells, indicating a reduction of the cell surface expression of all these molecules in the cells that overexpress MeCP2. This effect appears to be specific for these molecules since the level of transferrin receptor was basically unaffected by MeCP2 overexpression. The reproducibility and statistical significance of these observations was ascertained by repeating this experiment many times, which also allowed us to conclude that MeCP2 overexpression results in a similar 40% decrease of all three MHC class I molecules and of *β*-2-microglobulin (Figure 1B). In the experiment shown, the mock-transfected cells were simply treated with the transfection reagent, but similar results were obtained when the negative control consisted of empty plasmids, or plasmids expressing other proteins such as GFP (not shown).

No functional difference has been described between the two isoforms of MeCP2, MeCP2*A* and MeCP2*B*. We therefore explored whether these two isoforms had the same effect on MHC class I molecules and whether their effects might differ according to the cell type in which they are expressed. We transfected vectors encoding the Myc-tagged version of human MeCP2*A* or MeCP2*B* into either N2A or the mouse fibroblast cell line NIH 3T3. In this experiment, we detected MeCP2 expression by using an anti-Myc9E10 monoclonal antibody because it produced a much stronger signal than the anti-MeCP2 antibody. The level of MHC class I was evaluated at the same time by using a rat anti-pan-MHC antibody, M1/42. The double staining in mock-transfected and transfected cells was analysed by flow cytometry. MeCP2 overexpression induced a similar extent of repression of MHC class I irrespective of the isoform or cell type in which it was expressed (Figure 1C): the cell surface level of MHC class I decreased by approximately 35% when compared to the basal level (*p*<0.01 in N2A; *p*<0.001 in NIH 3T3) when either MeCP2*A* or MeCP2*B* were overexpressed in N2A or in NIH 3T3 cells.

We noted a correlation in these experiments between the repressive effect exerted by MeCP2 on MHC class I and *β*-2-microglobulin and the quantity of MeCP2 expressed by the transfected cells: cells expressing high levels of MeCP2 (dotted circle, Figure 1D) had less MHC class I on their surface than cells expressing MeCP2 at lower levels (continuous circle), which expressed levels of MHC class I similar to those in cells in which no MeCP2 staining was detected and to those in mock-transfected cells. Repression of MHC class I expression by MeCP2 therefore appears to require very high levels of MeCP2. We generated stably transfected lines of N2A expressing either MeCP2*A* or MeCP2*B* but the levels of protein expressed by these cells was relatively low (at best comparable to those in the continuous circle in Figure 1D). It is therefore of little surprise that the levels of MHC class I expressed by those cells was not discernibly different from that of untransfected cells (not shown). Evaluation by quantitative western blot of the levels of MeCP2 protein expressed in these various transfected cells revealed bands of comparable intensities in stable transfectants and in brain extracts, and bands that were two to five fold more intense in transiently transfected populations (not shown). Since mature neurons represent approximately 15% of all cells in the brain parenchyma, we can further estimate that our stable transfectants express levels of MeCP2 which are roughly one sixth of those found in mature neurons. Conversely, since the efficiency of the transient transfections in this experiment was approximately 10%, we estimate that the levels of MeCP2 attained in high expressors after transient transfections are 20 to 50 fold higher than the levels in stable transfectants (and therefore three to eight fold higher than in intra-cerebral neurons).

### Overexpression of MeCP2 markedly reduces MHC class I upregulation by IFN-γ

**Figure 2:**
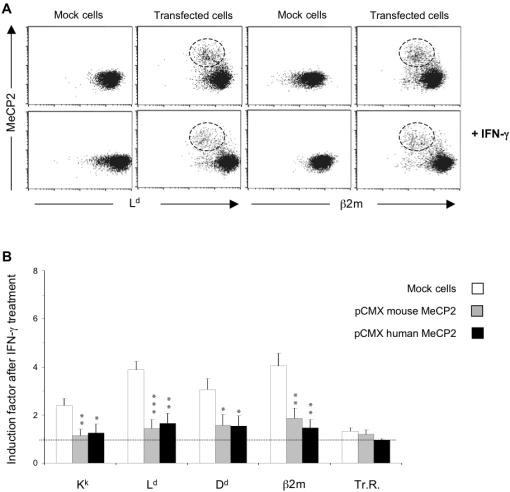
Transient overexpression of MeCP2 inhibits MHC class I induction by IFN-γ. N2A cells transfected with pCMX vectors expressing murine or human MeCP2 were treated or not with IFN-γ for 48 hrs and then double immunostained for cell surface β2-microglobulin, transferrin receptor or MHC class I and intracellular MeCP2. Panel A: Dot-plots of transfected cells analysed by flow cytometry showing the cell surface level of the MHC class I molecule L^d^ or β2-microglobulin (x-axis) plotted against the level of intracellular MeCP2 (y-axis). Panel B: The induction factor was calculated as the ratio of MFI of treated cells (over-expressing MeCP2 or untransfected cells) on MFI of untreated N2A cells. Values used for the histogram are the mean (±SEM) of induction factors obtained in seven independent transfection experiments. Statistical significance of difference between groups was analysed by using an unpaired t-test (*, *p*<0.05; **, *p*<0.01; ***, *p*<0.001).

The cytokine IFN-γ transactivates MHC class I expression predominantly by binding to IFN regulatory factor-1, which binds, in turn, to the interferon-stimulated response element, ISRE, a GC-rich region and therefore a potential binding site also for MeCP2 [47]. Since MeCP2 appeared to act as a repressor of MHC class I expression (Figure 1), we wanted to test whether it would interfere with transactivation of MHC class I expression by IFN-γ. To do so, we transfected N2A cells with pCMX vectors driving the expression of either murine or human forms of MeCP2*A*. The following day, these transiently transfected cells were divided into two flasks that were either treated or not with IFN-γ for 48 hrs. The levels of cell-surface MHC class I, *β*-2-microglobulin and transferrin receptor, and intracellular MeCP2, were evaluated by flow cytometry as in the previous experiments (Figure 2). As can be seen by the shift of the clouds between the higher and the lower panels, treatment with IFN-γ resulted in a two- to four-fold induction of MHC class I and *β*2-microglobulin in both mock-transfected populations and transfected populations (Figure 2A). Individual transfected cells expressing MeCP2 at high levels, however, had little more L^d^ and *β*2-microglobulin when treated with IFN-γ than untreated MeCP2-expressing cells (compare the populations encircled in Figure 2A IFN-γ with those without IFN-γ). Cells overexpressing MeCP2 therefore appear to respond to IFN-γ by upregulating MHC class I to a much lesser extent than do cells not expressing MeCP2.

The statistical significance of the differences in response to IFN-γ was confirmed for all three MHC class I molecules expressed by the N2A cell line (K^k^, L^d^, D^d^ ) and for *β*-2-microglobulin in six independent experiments (Figure 2B), whereas the levels of transferrin receptor were not significantly affected. This effect was also observed in other cell lines such as NIH 3T3 fibroblasts and human HEK 293 cells (not shown).

### MeCP2 mutants retain their repressive effect on MHC class I expression

**Figure 3:**
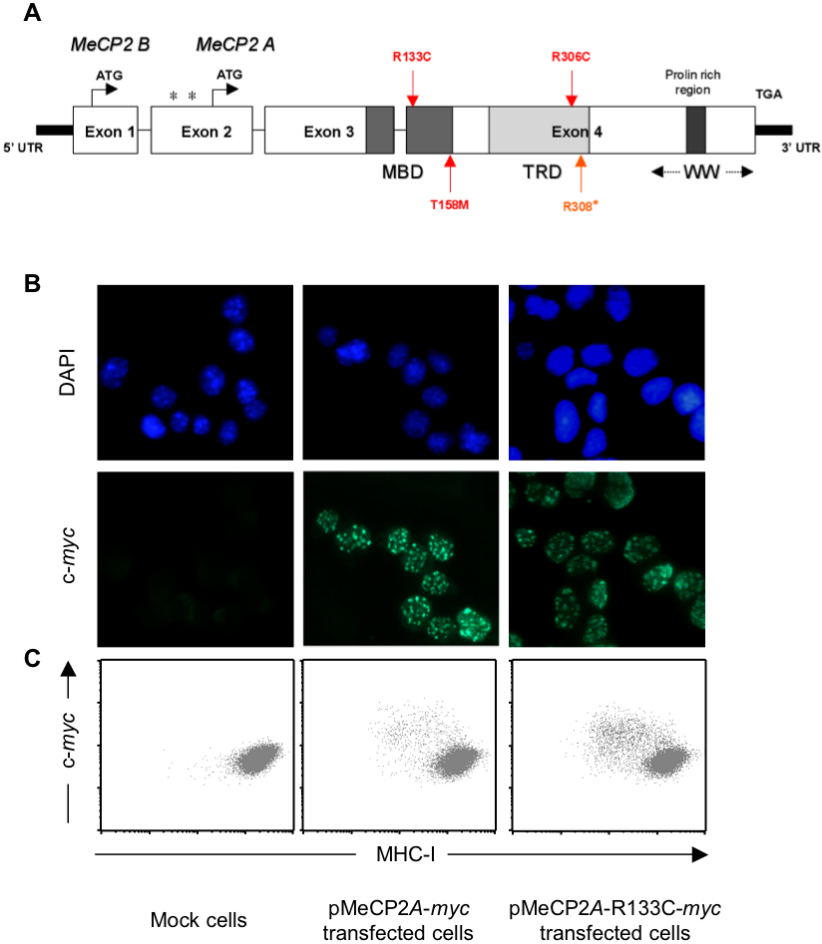
Mutant forms of MeCP2 that cause RTT retain their repressive effect on MHC class I expression. Panel A: Schematic representation of the MeCP2 protein. Red and orange arrows indicate the positions of the mutations introduced in MeCP2 by site-directed mutagenesis (MBD: methyl-CpG binding domain, TRD: transcription repression domain, WW: group II WW-domain-binding region). Panel B: N2A cells transfected with empty pcDNA3.1 (mock cells), with pcDNA3.1 expressing Myc-tagged MeCP2*A* (pMeCP2*A*-*myc*) or with pcDNA3.1 expressing Myc-tagged MeCP2*A* with the R133C point mutation (pMeCP2*A*-R133C-*myc*) were stained with mouse anti-Myc 9E10 monoclonal antibody, and FITC-labelled anti-mouse IgG antibody. Coverslips were mounted in DAPI-containing ProLong Gold antifade reagent (Molecular Probes) before observation by fluorescence microscopy. Panel C: N2A cells transfected as in panel B were double immunostained for cell surface MHC class I and intracellular Myc-tagged MeCP2, then analysed by flow cytometry. Similar data were obtained for all four mutated forms of MeCP2*A* and MeCP2*B* (not shown), and these observations were reproduced in three independent transfection experiments.

Many disease-causing mutations of *MECP2* have been described [48]. Among them, some occur more frequently than others, and/or have been more thoroughly characterised. To investigate the effect of these *MECP2* mutations on MHC class I expression, we transiently transfected the N2A cell line with plasmids expressing well-characterised mutants of both the A and B isoforms of MeCP2 (T158M, R133C, R306C and R308*). The point mutations T158M, R133C and R306C are located in the functional MBD and TRD domains of the protein (Figure 3A). The mutant form that is truncated after the R308 residue corresponds to the form of MeCP2 found in the mouse model of RTT generated by Dr. Zoghbi's group [13]. We performed mutagenesis on vectors expressing either the A or B form of Myc-tagged MeCP2. All the mutated plasmids were sequenced and checked for functional expression and intracellular localisation of the wild-type and mutated MeCP2 proteins by anti-Myc immunofluorescence on transiently transfected N2A cells (Figure 3B). All the mutant forms of Myc-taged MeCP2 were located in intranuclear punctate structures typical of the wild-type protein, which forms foci on heterochromatin [22], [49] (data is shown for the R133C MeCP2*A*-Myc mutant and the wild-type pMeCP2*A*-Myc protein only). Subsequently, we evaluated the cell-surface expression level of MHC class I in the transiently transfected N2A cells by flow cytometry using the rat anti-pan-MHC I antibody M1/42, as before. The intracellular MeCP2 level was evaluated based on the intensity of immunostaining for the Myc tag, and was found to be similar to the wild-type for both the A and B forms of the four mutants. The R133C MeCP2*A*-Myc mutant had the same effect as its wild-type counterpart on the cell-surface level of MHC class I (Figure 3C), whereas neither mutant nor wild type had a significant effect on expression of the transferrin receptor (not shown). Similar effects were found for both A and B isoforms of all four mutants tested (Data for T158M R306C and R308* are not shown). Thus, these mutations responsible for RTT do not abolish the repressive effect of MeCP2 on MHC class I in cells in culture. These results strongly suggest that the repressive function of MeCP2 on the levels of MHC molecules expressed by transiently transfected cells in vitro is unlikely to be directly related to the pathogenesis of Rett syndrome.

### Expression of MHC class I in cells from MeCP2-knockout mice is no different to that in cells from wild-type mice

**Figure 4:**
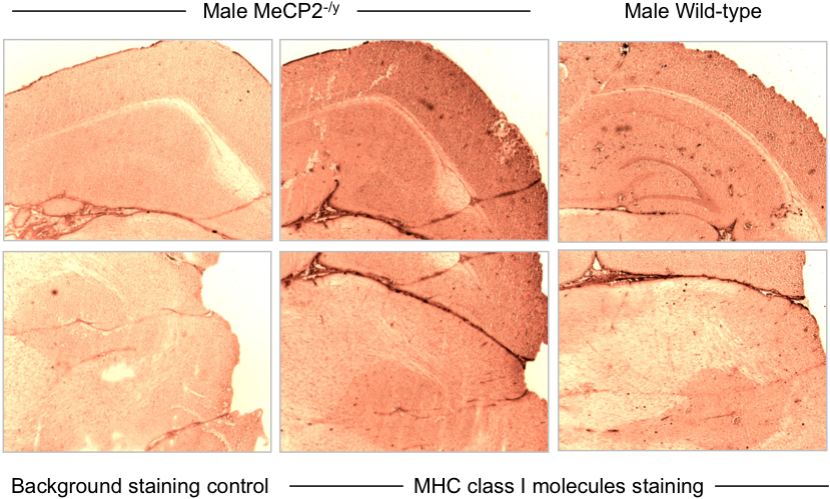
Evaluation of MHC class I expression in adult mouse brain slices. Serial frozen sections of adult male wild-type and MeCP2^−/y^ littermates were analysed for expression of MHC class I by immunohistochemistry using the rat R1-21.2 monoclonal antibody and EnVision detection technology (Dako). For the negative control, the same staining process was used omitting the primary antibody. Similar results were obtained with the M1/42 monoclonal antibody. Similar results were obtained in independent experiments on brains from three different pairs of mice.

Our data from experiments with cells in culture (above) suggest that the genes encoding MHC class I and β2-microglobulin are controlled by MeCP2; overexpression of normal MeCP2 downregulates their expression. To find out whether this is the case in vivo, we investigated whether neuronal cells from MeCP2 knockout mice (MeCP2^tm1.1Bird^) contained elevated levels of MHC class I by performing immunohistochemistry on frozen brain sections of MECP2 knockout hemizygous male (-/y) mice and of their wild-type littermates using two different rat monoclonal antibodies directed against mouse MHC class I molecules. The results we obtained suggest that there are slightly higher levels of MHC class I expression in some regions of the brains of MeCP2 knockout mice than in the same regions of the wild-type control brains (Figure 4). Although these differences were not always seen for all brain areas of MECP2 knockout mice compared to their control littermates, when a difference was seen, it was always for higher expression in MECP2 knockout animals.

**Figure 5:**
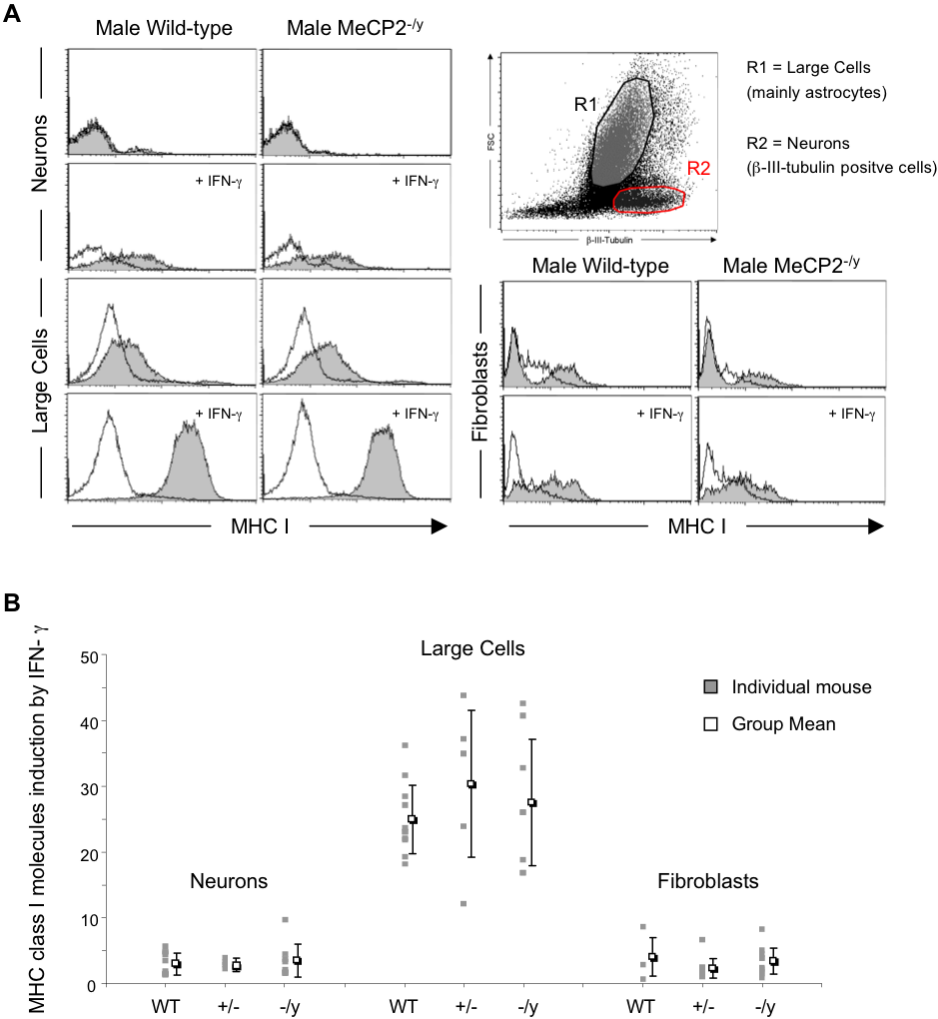
Deletion of MECP2 does not affect basal or IFN-γ -induced MHC class I expression in primary cultures of mixed glial cells. Mixed glial cell cultures established from two-day old wild-type, MeCP2^+/−^ and MeCP2^−/y^ mice (10, 6 and 8 animals per group, respectively) were treated or not with IFN-γ on the second day of culture and analysed two days later by flow cytometry for MHC class I expression. Neurons were identified by their intracellular staining with an anti-β-III-tubulin antibody (inset). Large cells, containing mainly astrocytes, were analysed separately by an appropriate forward/side scatter gate. Primary spleen fibroblasts from the same mice were also subjected or not to IFN-γ treatment and stained for their MHC class I expression. Panel A: Representative histograms showing cell surface staining (x axis) against cell number (y axis), obtained with cells from wild-type and MeCP2^−/y^ male littermates. White-filled curves represent background staining, gray-filled curves represent MHC I-specific staining. Panel B: MHC class I fold-induction in response to IFN-γ was calculated as the ratio of MFI of treated cells (induced MHC I level) on MFI of untreated cells (basal MHC I level). Grey-filled squares show MHC class I fold-induction for individual mice and for each cell type. White-filled squares represent the group's mean of fold-inductions (±SD).

The immunohistochemistry approach did not allow us to quantify the small variations we observed in MHC class I expression between MeCP2 knockout and wild-type mice or to identify the cell types that expressed MHC class I in the absence of MeCP2 (neurons, astrocytes, oligodendrocytes or endothelial cells). We therefore decided to look at primary cultures of brain cells (called mixed glial cells; MGCs) and fibroblasts from spleen taken from individual 2-day-old mice born from crossing a heterozygous female (MeCP2^tm1.1Bird +/−^) with a wild-type male. The tail DNA from each newborn mouse used to prepare the cell lines was analysed to establish the genotype of each culture. On the second day of culture, the MGCs were treated with IFN-γ or not and then analysed two days later for expression of MHC class I on the cell surface by flow cytometry (Figure 5). The fibroblast cultures, which took a few more days to establish, were similarly treated with IFN-γ after five days and analysed on the seventh day. Figure 5A shows typical examples of histograms obtained with wild-type and MeCP2^tm1.1Bird −/y^ male littermates. Similar data were obtained for all four genotypes: wild-type female (+/+) and male (+/y), heterozygous mutant female (MeCP2^tm1.1Bird −/+^) and hemizygous mutant male (MeCP2^tm1.1Bird −/y^).

Neurons, which we identified by their expression of *β*III-tubulin and their smaller size (see population R2, inset Figure 5A), had no detectable MHC class I (grey-filled curves) when compared to background (white-filled curves), and treatment with IFN-γ induced a small amount of MHC class I in cells from both the wild-type and knockout animals. Astrocytes, which comprise the majority of the population of large cells [50] (population R1, inset Figure 5A), spontaneously expressed more MHC class I, which was highly induced by IFN-γ but no significant differences were seen between the two genotypes. Although the populations in the cultures of spleen-derived fibroblasts were much more heterogeneous, similar levels of MHC class I were found in the cells from the wild-type and knockout animals. These measurements were performed on cell cultures derived from 24 newborn animals from nine independent litters, with very similar results.

To compare data obtained in independent experiments, we plotted the fold-induction of MHC class I expression after treatment with IFN-γ compared to untreated cells. Figure 5B displays the fold-induction of MHC class I by IFN-γ in each cell culture derived from an individual mouse as well as the mean for cell cultures of each genotype. No significant differences were found between wild-type and MeCP2-knockout cells. The deficiency in MeCP2 thus affects neither the basal nor the inducible MHC class I expression level in primary MGC cultures.

## Discussion

Reports by Shatz and colleagues [40], [41], [51] have demonstrated clearly that tight regulation of MHC class I genes in the CNS contributes to the establishment and maintenance of neuronal connections during development, as well as to plastic remodelling in the hippocampus and to neuronal signalling in specific areas of the brain [40], [41], [51]. We reasoned that the transcriptional repressor MeCP2 might be involved in regulating MHC class I expression in the CNS for three main reasons. Firstly, expression of MeCP2 is strictly regulated during development [52]–[54] and specifically in various cell types [55] and is highest in mature neurons [56], [57], which do not express MHC class I. Secondly, MeCP2 binds to methylated cytidine residues on CpG dinucleotides [21] and the genes for MHC class I are particularly rich in C/G residues [39]. Thirdly, mutations in the *MECP2* gene are responsible for the neurodevelopmental disorder RTT. We hypothesised that MeCP2 represses expression of MHC class I and that mutations responsible for RTT might cause the loss of this activity.

In support of the first part of this hypothesis, we showed clearly that overexpression of either of the two isoforms of MeCP2 by transient transfection of neuronal and fibroblastic cell lines results in reduced levels of MHC class I and *β*2-microglobulin at the cell surface. Furthermore, overexpression of MeCP2 blocked induction of MHC class I in these cells in response to IFN-γ. We could not determine whether this decreased expression of MHC class I and *β*2-microglobulin in response to MeCP2 overexpression was due to reduced transcription, translation or transport to the cell surface because the cells were transiently transfected; only cells that expressed high levels of MeCP2 showed reduced levels of MHC class I on their surface, so we could not quantify the levels of mRNA by RT-PCR or northern blot.

The fact that we observed very similar extents of repression of all three MHC class I forms (K^k^, L^d^ and D^d^) as well as of *β*2-microglobulin suggests that MeCP2 probably does not act directly on the promoters of the various loci encoding MHC class I, which are not identical. These considerations lead us to think that the effect of MeCP2 may be a more ‘global’, indirect mechanism than transcriptional repression by binding to methyl cytosine in the promoters.

The global decrease in cell surface MHC class I in MeCP2-expressing cells might be due to an effect on chromatin architecture encompassing the whole MHC region, which includes, in addition to the MHC class I loci, other genes involved in the assembly and transport of MHC molecules [58], [59] that might contribute to a global effect. If MeCP2 condenses the chromatin in the MHC region, this might block access of transcription factors and regulatory factors to the genes, as suggested by the work of Georgel and colleagues [30]. Such chromatin condensation would also explain the failure of IFN-γ to induce MHC class I in cells overexpressing MeCP2 if it prevented IFN regulatory factors from binding to their response element.

Our findings that the MeCP2 forms mutated either in the MBD or TRD domains conserve their repressive effect on MHC class I expression strengthen this interpretation that MeCP2 has an indirect effect on transcription through its effect on chromatin architecture. These mutations affect only transcriptional repression by MeCP2, preventing it from binding to methylated DNA and from recruiting repressor partners, but the other functions of the protein are not affected, particularly its capacity to silence gene expression by driving chromatin condensation [30], [60] and stabilising large silencing chromatin loops [31]. The ability of MeCP2 to condense chromatin by methylation-independent DNA binding relies on regions within the N-terminal 294 residues, distinct from the MBD [30], [61].

MeCP2 induces chromatin condensation in three successive steps: first, it binds to the linker DNA between nucleosomes; second, it brings the nucleosomes together in a ‘stem conformation’ through DNA–protein interactions, and third, it binds to the nucleosomes themselves to produce full chromatin compaction [60]. Whereas the C-terminal region of MeCP2 is dispensable for the two first steps, it is apparently required for maximal compaction of the chromatin since the R294X mutation abolishes bridging between nucleosomes [60], [61]. By contrast, the R133C mutation, which abolishes the selective recognition of methylated DNA, retains the chromatin compaction properties of the wild-type protein [30]. The two other point mutations we tested in this study, T158M and R306C, like R133C, may retain their compaction properties and thus their repressive effect on MHC genes. Our findings with the truncated form R308*, which also retains the same repressive effect on MHC class I expression as the wild-type protein, indicate that the repression of MHC genes by MeCP2 does not involve the C-terminal portion of the protein. The effects of MeCP2 on MHC expression therefore probably does not involve the third step of MeCP2-induced chromatin condensation (above) but may involve a conformational change in the ‘stem organization’ of the chromatin around the MHC region [60], [61].

If MeCP2 represses MHC class I expression, we expected to see elevated levels of MHC class I in the brains of *MECP2* knockout mice (strain MeCP2^tm1.1Bird^) when compared to mice with fully functional *MECP2* [11]. When we stained brain slices immunohistochemically with two different antibodies, we saw a small but reproducible increase in MHC class I expression in the brains of three knockout male mice when compared to those of their wild-type littermates, but these results were purely qualitative, and it was not possible to quantify these differences by immunohistochemistry. We decided, therefore, to produce primary cultures of MGCs from knockout mice in order to quantify MHC class I expression by flow cytometry. This approach also allowed us to distinguish a neuronal population, comprised of small cells expressing *β*III-tubulin, and a population of large cells comprised mostly of astrocytes. This quantitative analysis detected no difference between MHC class I levels on the surface of cells derived from animals carrying an inactivated *MECP2* gene and those from their wild-type littermates whether for the basal level of MHC class I expression or for the level induced by IFN-γ. This was true for the neuronal population and the astrocyte population, as well as for primary cultures of spleen fibroblasts.

We conclude from these studies that there may be a small quantitative increase in MHC class I in the brains of *MECP2* knockout mice when compared to the wild type but this increase is not seen in isolated primary MGCs containing mostly neurons and astrocytes. The possibility remains that the levels of MeCP2 in these primary cell cultures were insufficient to see a difference. Indeed, our experiments with N2A cells showed that relatively high levels of MeCP2 are necessary to observe its repressive effect on MHC class I expression. Several studies have shown a direct correlation between age and MeCP2 expression, with maximal levels of expression in post-mitotic mature and differentiated neurons [52], [53], [55]–[57], [62]. Although the neuronal populations in our MGCs expressed *β*III-tubulin, a marker of neuronal differentiation, they were very probably far from being fully mature. In support of this, we were unable to detect MeCP2 expression in neonatal MGCs by flow cytometry (not shown). The neurons that we analysed in neonate MGC might therefore have been too immature to demonstrate a defect in MHC regulation due to MeCP2 deficiency. It was, however, not feasible to obtain brain tissue from adult *MECP2* knockout animals for these studies because the mice die at around eight weeks old.

Three groups have performed transcriptome analyses on microarrays of cDNA prepared from post-mortem brain tissue of girls with RTT and from the brains of *MECP2* knockout mice [63]–[65]. At least one of these studies supports the idea that MeCP2 may influence MHC expression in the CNS: Colantuoni *et al.* [63] found a 5.8-fold increase in expression of the mRNA for the MHC class I molecule HLA-A in a RTT syndrome patient relative to matched controls. This small increase in MHC class I expression in the absence of MeCP2 is consistent with our hypothesis and further suggests that such subtle disregulation might be detectable only in older mice than those we used in our study.

We found that the forms of MeCP2 carrying RTT-causing mutations retained their repressive function on MHC expression in transiently transfected cells. Although this observation does not go against our findings that MeCP2 can down-regulate MHC expression in cells where it is expressed at high levels, these results lead us to conclude that repression of MHC class I expression by MeCP2 is very probably not directly relevant to the pathogenesis of RTT. Mutations in the *MECP2* gene are also suspected to be at the origin of a large panel of other neurological diseases such as autism [66], [67], Angelman syndrome [68], X-linked mental retardation [69], [70], and severe neonatal encephalopathy [71], [72]. It may therefore be that those types of mutations responsible for other diseases could be related to the MHC-regulating activity of MeCP2. Additionally, the majority of *MECP2* mutations in RTT are loss-of-function mutations, but overexpression of *MECP2* by gene duplication in a mouse model [73], as well as in human clinical cases [74]–[77], also causes mental retardation and progressive neurological diseases. Since high levels of MeCP2 can repress cell surface expression of MHC class I, at least in neuronal cell lines, MeCP2 overexpression might result in an overrepression of MHC gene expression in the CNS, which may contribute to certain pathologies by preventing MHC molecules from fulfilling their roles during CNS development and in synaptic plasticity [51]. Inappropriate temporal and spatial repression of MHC genes by overexpressed MeCP2 might induce defects in neuronal functions similar to those observed in *β*2m/TAP1 deficient mice [41]. Further experiments will be required to explore this eventuality.

This leaves us with the important conclusion that high levels of MeCP2, like those found in mature neurons, repress expression of MHC class I molecules and this may be an important physiological factor contributing to the repression of MHC class I in the CNS.

## Materials and Methods

### Mice

The *MEPC2* knockout mice (MeCP2^tm1.1Bird^) were obtained from the *Institute for Stem Cell Research*, Edinburgh, UK. These mice have a RTT-like progressive neurodevelopmental disease very similar to that seen in patients; the males (−/y) suffer from a much more severe form of the disease than the heterozygous (+/−) females, and are sterile. MeCP2^+/−^ female mice were mated with C57BL/6 male mice purchased from the Centre de Recherche et d'Élevage, Janvier, France. The litters obtained were genotyped initially as recommended by the Jackson Laboratory, and later with an optimised set of primers [78]. All experiments involving animals were performed in compliance with the relevant laws and institutional guidelines.

### Cell lines

The N2A and NIH 3T3 cell lines were maintained in DMEM with 10% foetal calf serum (FCS) and antibiotics. For the origin and description of these cell lines see ATCC.

### Antibodies

Polyclonal primary antibodies: rabbit anti-MeCP2 polyclonal IgG (Upstate), mouse anti- C terminal peptide of MeCP2 (Sigma) and rabbit anti-beta III-tubulin polyclonal IgG (Abcam).

Hybridoma supernatants from clones (obtained from ATCC): 9E10 (mouse anti-myc IgG1), M1/42 (rat anti-H2 IgG_2a_), R1-21.2 (rat anti-mouse H2 IgG_2b_), HB25 (mouse anti-H2 K^k^ IgG_2a_), 28.14.8 (mouse anti-H2 D^b^, L^d^ and D^q^ IgG_2a_), 30.5.7 (mouse anti-H2 L^d^, D^q^ and L^q^ IgG_2a_), 34.4.20 (mouse anti-H2 D^d^ IgG_2a_), Y-3 (mouse anti-H2 K^b^ IgG_2b_), S19.8 (mouse anti-beta 2 microglobulin b and c IgG_2b_), R17.217 (rat anti-mouse transferrin receptor IgG_2a_).

Secondary fluorescent antibodies: FITC goat anti-mouse IgG (Dako), fluorescein goat anti-rabbit IgG (H+L) (Molecular Probes, Invitrogen), AlexaFluor 647 goat anti-rat IgG (H+L) (Molecular Probes, Invitrogen), AlexaFluor 647 and 680 goat anti-mouse IgG (H+L) (Molecular Probes, Invitrogen).

### Plasmids

pCMX vectors expressing mouse MeCP2α, MeCP2β and human MeCP2*A* were described previously [18], [79]. pcDNA3.1(A) vectors expressing Myc-tagged human MeCP2*A* and MeCP2*B* [19] were provided by Dr. Berge A. Minassian of the Hospital for Sick Children, Toronto, Ontario, Canada.

### Mutagenesis

Site-directed mutagenesis of pcDNA3.1(A) vectors driving expression of Myc-tagged human MeCP2*A* and MeCP2*B* was carried out as described previously [80]. Briefly, during a first PCR reaction typically [9× (95°C for 30 s, 55°C for 1 min, 68°C for 1 min)] using high-fidelity Taq polymerase (Invitrogen), a DNA template was generated between a forward T7 oligonucleotide (5′-GTAATACGACTCACTATAG-3′) annealing to pcDNA3.1 (A) upstream of the multiple cloning site and a reverse oligonucleotide annealing to the MeCP2 sequence and carrying the chosen mutations:

- R133C: 5′-aatcaactccactttactgcagaaggcttttccctg-3′
- T158M: 5′-ccctctcccagttaccatgaagtcaaaatcatt-3′
- R306C: 5′-gatgctgaccgtctcacgcgtcttgcacttcttgatggggag-3′

These primers were designed to produce or remove a restriction enzyme site allowing simple screening of the recovered plasmids (gain of an PstI site for R133C; gain of an NlaIII site for T158M, and loss of an SmaI site for R306C). During a second PCR reaction [9× (95°C for 30 s, 68°C for 6 min)], the newly amplified DNA fragments served as megaprimers to complete the synthesis of the remainder of the plasmids. The extension time of the last cycle was 16 min, followed by digestion with 10 U *Dpn*I at 37°C for 1 hr to destroy the original methylated plasmids. The mutated plasmids were recovered by transforming competent DH5α bacteria and they were screened by restriction digest. The sequence of the inserts corresponding to the MeCP2 open reading frame was then checked by direct sequencing.

To generate pcDNA3.1 plasmids expressing the R308* truncated forms of Myc-tagged MeCP2*A* and MeCP2*B*, a PCR reaction [12× (94°C, 45 s; 50°C, 45 s; 68°C, 2 min 30 s), 12× (94°C, 45 s; 68°C, 2 min 30 s)] using high-fidelity Taq polymerase (Invitrogen), was carried out on pcDNA3.1-MeCP2*A* using the following oligonucleotides:

- Forward T7 primer: 5′-GTAATACGACTCACTATAG-3′
- Reverse primer: 5′-gcgtctagagagggtggacaccagca-3′

The reverse common primer, annealing in the *MECP2* sequence, was designed to include an XbaI restriction site immediately following residue 308 of MeCP2, which is also located upstream of the sequence encoding the Myc tag in the original pcDNA3.1 Myc plasmid. The amplified MeCP2(1–308) sequence was digested with XcmI and XbaI and ligated, using T4 DNA ligase (NEB), into pcDNA3.1 human MeCP2*A* or *B* myc-tagged plasmids digested with the same enzymes. The plasmids expressing truncated Myc-tagged MeCP2*A* and *B* forms were recovered by transforming competent DH5α bacteria and they were screened by restriction digest. The sequence of the inserts corresponding to the MeCP2(R308*) open reading frame was then checked by direct sequencing.

### Transfection

Transfections were carried out using FuGENE 6 (Roche Applied Bioscience), following the manufacturer's instructions (using 2 µg of plasmid and 6 µl of transfection reagent). Briefly, N2A or NIH 3T3 cells were plated in 25 cm^2^ flasks at least 12 hrs before transfection. Stable N2A transfectants expressing Myc-tagged wild-type and mutated MeCP2*A* and MeCP2*B* were selected by adding 0.5 mg/ml of G418 three days after transfection, and the populations obtained were subsequently maintained in this selecting medium.

### Quantitative Western Blot

Whole cellular extracts from adult mouse brain, and from transiently and stably transfected N2A cells were prepared, and blotted on nitrocellulose after separation by 4–12% SDS-PAGE (50 µg of cellular extract per sample). Levels of MeCP2 were then measured on an Odyssey infrared scanner (Li-Cor) after staining with a mouse anti-MeCP2 polyclonal antibody (C terminal portion, Sigma), followed by an Alexa 680 anti-mouse secondary antibody. Normalisation between samples was carried out using a mouse monoclonal against GAPDH (ab9484, abcam) and the same secondary reagent on the lower part of the same blot.

### Cells staining and cytometry

For immunochemical staining of the cell surface for MHC class I, *β*-2-microglobulin or transferrin receptor, cells were harvested by trypsinisation and washed once with medium to obtain a single-cell suspension. The resuspended cells were then incubated on ice with a saturating concentration of the appropriate hybridoma supernatant. Thirty minutes later, cells were washed three times in phosphate-buffered saline (PBS) containing 2% FCS, then incubated for thirty minutes on ice with the suitable secondary reagent and washed three times before fixation in PBS containing 1% paraformaldehyde. When intracellular MeCP2 or *β-*III-tubulin was also analysed, further staining steps were performed as described above, but adding 0.3% saponin in all buffers. Labelled cells were analysed using a FACScalibur cytometer and CellQuest software (BD Biosciences). Dead cells were excluded using appropriate forward/side scatter gates.

### Immunohistochemistry of brain slices

Brains from adult MeCP2^−/y^ and wild-type mice were embedded in Tissue-Tek OCT compound (Sakura) and frozen in dry ice-cold isopentane. Serial coronal sections of 10 µm were cut with a cryostat at −20°C, placed on Super Frost Plus slides (Menzel-Gläser), air dried overnight at room temperature and fixed in acetone and air dried rapidly. The tissue sections were then rehydrated in PBS containing 3% bovine serum albumin (BSA) and endogenous peroxidase activity was quenched by incubating with peroxydase block reagent (Dako). Following washes in PBS containing 1% BSA, the specimens were incubated with saturating concentrations of rat hybridoma supernatant for 1 hr and then rinsed in PBS containing 1% BSA before incubation with the rabbit anti-rat Ig antibody Z0494 (Dako). The sections were then treated with EnVision as instructed by the manufacturer (Dako) using a labelled polymer-HRP goat anti-rabbit Ig. Finally, slides were mounted in Mowiol. Observation and image acquisitions were carried out on a Leica RM-IRB microscope using the 10× objective. Pictures were taken with a COHU CCD camera.

### Immunofluorescence staining

N2A cells growing on coverslips were transfected with empty pcDNA3.1(+), with pcDNA3.1 expressing Myc-tagged wild-type MeCP2*A* or *B*, or pcDNA3.1 expressing Myc-tagged mutated MeCP2*A* or *B* containing the T158M, R133C, R306C or R308* mutations. Cells were fixed with acetone and incubated with mouse anti-Myc monoclonal antibody (9E10) followed by FITC-labelled anti-mouse IgG antibody for 30 min at room temperature. After the final wash, the coverslips were mounted in DAPI-containing ProLong Gold antifading reagent (Molecular Probes). Observation and image acquisition were carried out on a Leica RM-IRB microscope using 40× or 20× objectives. Pictures were taken with a COHU CCD camera and acquired as TIFF stacks of images with Q-Fluoro software, after integration of the signal for 1 sec for in the case of FITC labelling (no integration of the signal was necessary for DAPI labelling).

### Primary cultures

Brains removed from two-day old mice were cut in small pieces in ice-cold PBS containing glucose, then centrifuged at 80 *g* for 5 min at 4°C and digested with trypsin (Gibco) for 40 min at 37°C. The trypsin was blocked by adding DMEM supplemented with 10% FCS for 5 min at room temperature. The digested brain material was then washed twice with DMEM containing 10% FCS and once in neurobasal medium (Gibco) supplemented with B27 and 2% FCS, and recovered between washes by centrifugation at 80 *g* for 5 min at 4°C. During the final wash, mechanical dissociation of the cells into a single-cell suspension was achieved by pipetting up and down several times. Brain cell suspensions were plated on six-well plates coated at least 12 hrs before with poly-D-ornithine (SIGMA) and maintained in neurobasal medium containing B27 and 2% FCS.

For cultures of splenic fibroblasts, spleens were cut into small pieces in PBS/glucose, digested in trypsin and washed, as above, and finally plated on six-well plates and maintained in RPMI containing 10% FCS.
